# Not Everything Is as It First Appears

**DOI:** 10.3201/eid3011.AC3011

**Published:** 2024-11

**Authors:** Byron Breedlove

**Keywords:** Abraham Mignon, about the cover, Not everything is as it first appears, art–science connection, foodborne illnesses, food safety, Vibrio

**Figure:**
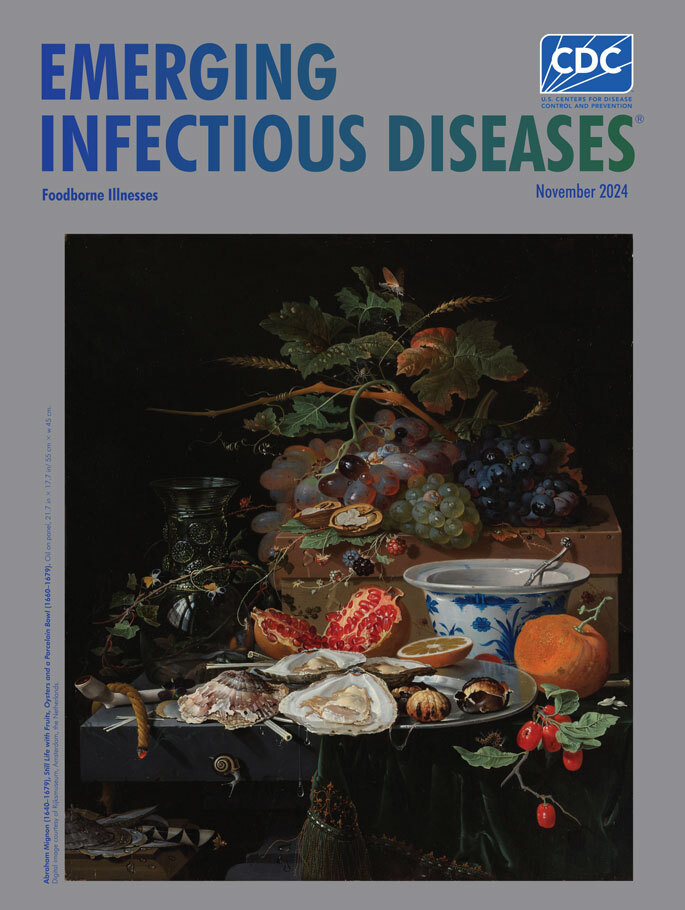
**Abraham Mignon (1640–1679). *Still Life with Fruits, Oysters and a Porcelain Bowl* (1660–1679).** Oil on panel, 21.7 in × 17.7 in/55 cm × 45 cm. Digital image courtesy of Rijksmuseum, Amsterdam, the Netherlands.

Although Abraham Mignon was born in Frankfurt, Germany, he is associated with the Dutch painters of his time. When Mignon was 9 years old, his parents, who were shopkeepers, arranged an apprenticeship with Jacob Marrel, a German still-life painter. Marrel had previously traveled to Utrecht, the Netherlands, to work and study, and he returned there in 1664 accompanied by Mignon, who was then 24.

In 1669, both artists became members of the Guild of Saint Luke, and for several years Mignon studied and worked with fellow guild member, Jan Davidsz de Heem, a Dutch still-life specialist. The Rijksmuseum states, “Mignon’s paintings of flowers and fruit feature the same opulent style of composition and the same brilliant colours as De Heem’s work.” Mignon further assimilated into Dutch society when he married Maria Willaerts in 1675, but the union proved short, as he died at age 39 in March 1679.

Throughout the 17th Century, still-life painting in the Netherlands flourished. Art historian Walter Liedtke wrote, “In general, the rise of still-life painting in the Northern and Spanish Netherlands (mainly in the cities of Antwerp, Middelburg, Haarlem, Leiden, and Utrecht) reflects the increasing urbanization of Dutch and Flemish society, which brought with it an emphasis on the home and personal possessions, commerce, trade, learning—all the aspects and diversions of everyday life.”

*Still Life with Fruits, Oysters and a Porcelain Bowl*, featured on this month’s cover, is among an estimated 400 paintings attributed to Mignon and exemplifies his eye for detail, precision, and texture. The Rijksmuseum, where the painting may be viewed, notes: “The composition of this still life is both sumptuous and inventive. Mignon set the roemer, the green glass at the left, upside down. Reflected in it is a window, and a view of a church tower in Utrecht, where Mignon lived. The porcelain bowl from Asia was a sign of prosperity as well as being a showpiece on the table.”

In the painting, the vivid colors and lifelike textures are enhanced by a black background. The canvas is crowded with resplendent grapes and berries, the top of a green pumpkin or gourd, a halved pomegranate, an orange slice, cherries hanging from a snippet of a branch, charred chestnuts, a split walnut, glistening raw oysters, and various leaves and vines. Smoke tendrils drift from tobacco stuffed into a white clay pipe and the glowing tip of a twist of rope used to light the pipe. Mignon’s expertise in creating textures is also shown by an ornate porcelain bowl, the hard-to-see inverted green drinking glass, or roemer, on the left, and drops and pools of a viscous liquid.

Symbolic and allegorical interpretations in still-life paintings from the Dutch Golden Age are abundant. Art historian Emily Snow wrote, “Grapes symbolize the themes of pleasure and lust associated with Bacchus, the Roman god of wine. Pomegranates are associated with Persephone, the Greek goddess of spring and queen of the underworld.” Snow explains, “Oysters were especially popular in still life paintings of the Dutch Golden Age and were not considered a luxury food at the time. Like other shells, they symbolize birth and fertility.”

Beneath its veneer of abundance, however, Mignon’s painting reveals a more complicated commentary. Entomologists (meant in the broadest sense of the historical term) could describe which moth, spider, and caterpillar have taken residence, and a malacologist could identify the snail exploring the side of the table. Several grapes have split or shriveled, and the ripe cherries have already fed the hungry caterpillar. One oyster shell is overturned on the table, and others are piled over an ornate harlequin-handled knife lying on a smaller table. All may be interpreted as symbols of decay and death, the transience of life and beauty.

Modern viewers, accustomed to hearing about outbreaks of foodborne diseases and recalls of various foods, might ponder whether Mignon’s colorful feast would be safe to eat. Could those grapes and berries have been grown near contaminated water or picked by people practicing poor hygiene? Might those oysters harbor some form of *Vibrio* bacteria that live in coastal waters and can contaminate shellfish with a bacteria or toxin?

Several articles in this issue of *Emerging Infectious Disease* address foodborne disease outbreaks. One reports food poisoning in Australia caused by *Vibrio parahemolyticus* in oysters. Another documents disease in Nigeria caused by *Vibrio cholerae*, a bacteria endemic to the environment there and a source of cholera through contact with contaminated food and water. A third recounts a prolonged outbreak of listeriosis in Switzerland, linked with a disease typically spread through contaminated dairy products, meat, fish, fruits, or vegetable—in this case, baker’s yeast products.

More than 200 various types of foodborne diseases have been identified. The Centers for Disease Control and Prevention reports that in 1 year in the United States, 48 million people will get sick from a foodborne illness, nearly 130,000 will be hospitalized, and 3,000 will die. The World Health Organization estimates, “Each year worldwide, unsafe food causes 600 million cases of foodborne diseases and 420,000 deaths.” The exact toll from foodborne illness is, however, not known.

Scallan, Hoekstra, and Angulo et al. noted in a 2011 research article in *Emerging Infectious Diseases* that “Estimates of the overall number of episodes of foodborne illness are helpful for allocating resources and prioritizing interventions,” but they also explained that various factors make it difficult to develop accurate estimates: many different agents can contaminate food (bacteria, viruses, parasites, and toxins), contact with animals or drinking contaminated water may cause illness, and foodborne pathogens have different effects on their hosts depending on the person’s age and overall health. They point out that “only a small proportion of illnesses are confirmed by laboratory testing and reported to public health agencies.”

Public health investigators often assess information that relies on the people’s memories. Responding to outbreaks of foodborne illnesses requires following clues, and as is the case with studying Dutch still-life paintings, requires an eye for detail, because not everything is as it first appears.
